# Sphingomyelin generated by sphingomyelin synthase 1 is involved in attachment and infection with Japanese encephalitis virus

**DOI:** 10.1038/srep37829

**Published:** 2016-11-28

**Authors:** Makoto Taniguchi, Takafumi Tasaki, Hideaki Ninomiya, Yoshibumi Ueda, Koh-ichi Kuremoto, Susumu Mitsutake, Yasuyuki Igarashi, Toshiro Okazaki, Tsutomu Takegami

**Affiliations:** 1 Department of Life Science, Medical Research Institute, Kanazawa Medical University, 1-1 Daigaku, Uchinada, Ishikawa 920-0293, Japan; 2 Histology Laboratory, Research Support Center, Medical Research Institute, Kanazawa Medical University, 1-1 Daigaku, Uchinada, Ishikawa 920-0293, Japan; 3 Department of Hematology and Immunology, Kanazawa Medical University, 1-1 Daigaku, Uchinada, Ishikawa 920-0293, Japan; 4 Graduate School of Arts and Sciences, University of Tokyo, Komaba, Meguro-ku, Tokyo, 153-8902, Japan; 5 Department of Advanced Prosthodontics, Graduate School of Biomedical & Health Sciences, Hiroshima University, Hiroshima 734-8553, Japan; 6 Department of Applied Biochemistry and Food Sciences, Faculty of Agriculture, Saga University, Saga 840-8502, Japan; 7 Laboratory of Biomembrane and Biofunction Chemistry, Faculty of Advanced Life Sciences, Hokkaido University, Kita 21-jo, Nishi 11-chome, Kita-ku, Sapporo 001-0021, Japan

## Abstract

Japanese encephalitis virus (JEV) is a mosquito-borne RNA virus which infects target cells via the envelope protein JEV-E. However, its cellular targets are largely unknown. To investigate the role of sphingomyelin (SM) in JEV infection, we utilized SM-deficient immortalized mouse embryonic fibroblasts (tMEF) established from SM synthase 1 (SMS1)/SMS2 double knockout mice. SMS deficiency significantly reduced both intracellular and extracellular JEV levels at 48 h after infection. Furthermore, after 15 min treatment with JEV, the early steps of JEV infection such as attachment and cell entry were also diminished in SMS-deficient tMEFs. The inhibition of JEV attachment and infection were recovered by overexpression of SMS1 but not SMS2, suggesting SMS1 contributes to SM production for JEV attachment and infection. Finally, intraperitoneal injection of JEV into SMS1-deficient mice showed an obvious decrease of JEV infection and its associated pathologies, such as meningitis, lymphocyte infiltration, and elevation of interleukin 6, compared with wild type mice. These results suggest that SMS1-generated SM on the plasma membrane is related in JEV attachment and subsequent infection, and may be a target for inhibition of JEV infection.

Sphingolipids such as sphingomyelin (SM), ceramide, and glycosphingolipids (GSLs) are essential constituents of cellular lipid-bilayer membranes and have roles in diverse cell functions by regulating cellular signalling pathways. These sphingolipids enable ligand-receptor signalling associated with cell proliferation, migration, cell death, and inflammation^1^. Recent studies showed viruses also utilize membrane sphingolipids at various steps of their life cycle including attachment and entry into target cells^2^,^3^. For example, human immunodeficiency virus (HIV) interacts with GSLs such as Gb3 or galactosylceramide via their receptors and achieves entry into cells^4^,^5^,^6^,^7^. Murine polyoma virus and simian virus 40 (SV40) use gangliosides as entry receptors^8^. Furthermore, rhinovirus accelerates ceramide-enriched platform formation and endocytosis^9^,^10^. In Ebola virus infection, viral attachment appears to be SM dependent^11^.

Japanese encephalitis virus (JEV) is a mosquito-borne flavivirus related to Dengue virus, West Nile virus, yellow fever virus, and tick-borne encephalitis virus^12^. JEV infects neuronal cells and causes severe encephalitis, which has a 30% mortality rate and causes neurological sequelae in 50% of survivors. JEV consists of a small enveloped particle (40–60 nm) and positive-sense RNA genome of approximately 11 kb. Its viral RNA encodes single large polyproteins; three structural proteins (capsid [C], pre-membrane [prM], and envelope [E]), and seven non-structural protein (NS1, NS2a/2b, NS3, NS4a/4b, and NS5). The structural proteins compose the viral particle, and the E protein (JEV-E) is important for interaction with cell surface receptor molecules and entry into target cells. Although multiple cellular components such as heat-shock cognate protein 70 (Hsp70)^13^,^14^, glycosaminoglycans^15^,^16^, and laminin^17^ have been associated with JEV infection, the exact mechanism and cellular target of JEV are largely unknown. Moreover, recent studies have complicated matters, with multiple mechanisms of JEV entry being reported: clathrin-dependent endocytosis in mosquito cells^14^ and porcine kidney epithelial PK15 cells^18^; a clathrin-independent pathway in mouse and human neuronal cell lines^19^; caveola-mediated entry in rat neuroblastoma cells^20^; and entry via lipid rafts in hepatoma Huh7 cells^21^ and neural stem/progenitor cells^22^. Notably, SM is expressed in clathrin-coated pits, caveolae, and lipid rafts, but its role in JEV attachment and infection remains unclear.

SM is catalysed from ceramide and phosphatidylcholine (PC) by SM synthases (SMSs) with diacylglycerol (DAG)^23^,^24^. SMS has three isoforms, SMS1, SMS2, and SMS-related protein (SMSr). SMS1 and SMS2 have SM synthesis activity, but SMSr is a ceramide-phosphoethanoramine synthase. SMS1 is localized in the Golgi apparatus, while SMS2 is present in both the plasma membrane and Golgi. Previously, we showed that SMS1, but not SMS2, contributes to the generation of plasma membrane SM and transferrin-mediated proliferation in mouse lymphocytes^25^. In contrast, both SMS1 and SMS2 have been shown to contribute to SM production related to cell growth, migration, and cell death in various cell types^23^. However, the contributions of SMS1 and/or SMS2 to SM production in viral infections have not yet been investigated.

In this study, we focus on the role of SM and SMS in JEV attachment and infection of target cells using transformed mouse embryonic fibroblasts (tMEFs) derived from SMS knockout (KO) mice, which have depleted SM levels. Both JEV infection of target cells and attachment to the cell surface were attenuated under SM-depleted conditions. In addition, induction of SMS1 but not SMS2 recovered JEV attachment and infection in SMS KO tMEFs. In line with these *in vitro* studies, SMS1-deficient mice exhibited a significant reduction of JEV infection and its associated pathologies in the brain. These results demonstrate that SM generated by SMS1 is implicated in JEV attachment and entry into target cells. Therefore, SM and SMS1 may be the target against JEV infection.

## Results

### SM and SMS are necessary for JEV infection

**Figure 1:**
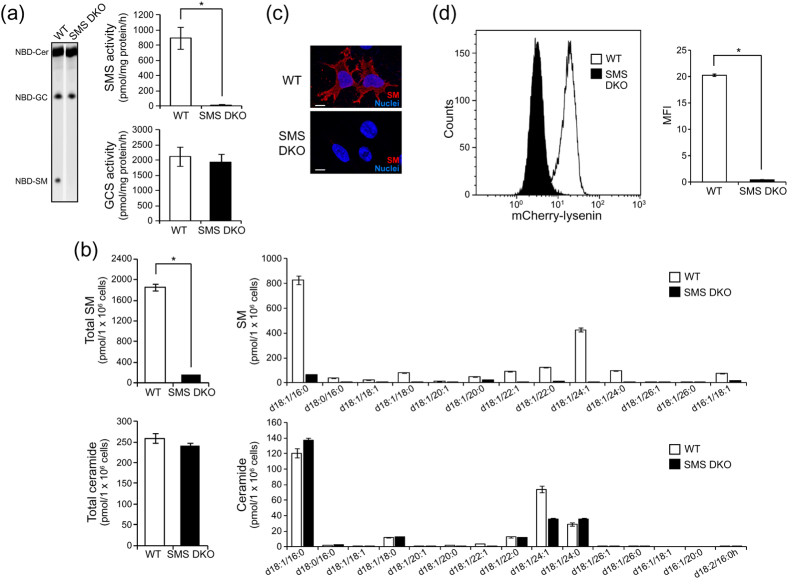
Characterisation of SMS knockout MEFs. (**a**) *In vitro* SMS and GCS activities were measured using C_6_-NBD-ceramide as the substrate as described in the Materials and Methods. Wild type (WT) and SMS double knockout (SMS DKO) tMEFs were harvested and homogenized. Lysate (100 μg) was added to the reaction solution and incubated for 1 h at 37 °C. Fluorescent lipids were extracted and quantified using a LAS-4000 fluorescent imaging system. The results are presented as the mean ± the standard deviation (SD) (n = 4). *P < 0.005. (**b**) SM and ceramide levels were assessed by LC-MS/MS. The value presented is the mean ± SD (n = 3). *P < 0.005. (**c**) SM was stained with MBP-conjugated lysenin. Then, nuclei were stained with Hoechst 33342, and the cells were observed by confocal microscopy. Scale bars, 10 μm. (**d**) Membrane SM was detected with mCherry-lysenin and flowcytometer. Mean fluorescence intensity (MFI) was measured by Kaluza software. The value presented is the mean ± SD (n = 3). *P < 0.005.

To clarify the role of SM in JEV infection, we utilized SM-deficient immortalized mouse embryonic fibroblasts (tMEFs) derived from SMS1 and SMS2 double knockout (DKO) mice^26^,^27^. As shown in Fig. 1a, SMS activity was decreased in SMS DKO tMEFs compared with MEFs established from wildtype mice (WT tMEFs). However, SMS deficiency had no effect on glucosylceramide synthase (GCS) activity. The levels of cellular SM and ceramide were measured by LC-MS/MS (Fig. 1b). According to the loss of SMS activity, cellular SM content was also reduced in SMS DKO tMEFs, but ceramide was unchanged (Fig. 1b). Because SM is the major sphingolipid constituent of the plasma membrane, we detected membrane SM with lysenin, a well-known binding protein of SM, using immunocytochemistry and flow cytometry. In WT tMEFs, SM was detected with lysenin on the cell surface (Fig. 1c and d). However, cell surface lysenin staining was significantly decreased in SMS DKO tMEFs (Fig. 1c and d). Thus, SMS DKO tMEFs had clearly reduced SM contents in plasma membrane.

**Figure 2:**
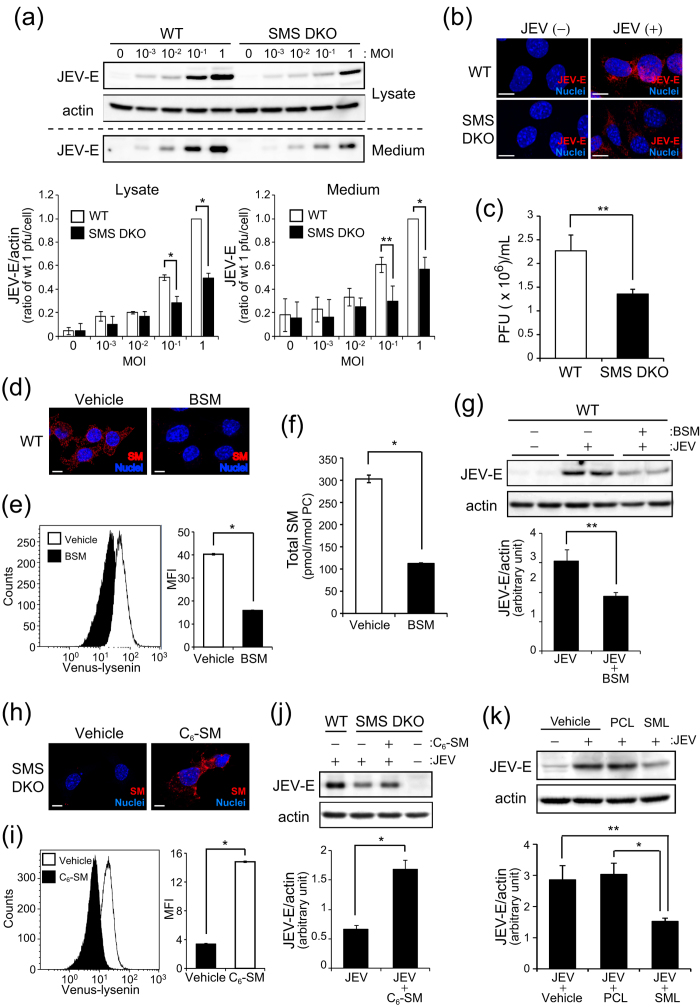
SMS deficiency blocks JEV infection. (**a**) The wild type (WT) and SMS knockout (SMS DKO) tMEFs were infected with JEV at mock or indicated concentration (MOI = 1 × 10^−3^ to 1) for 1 h in DMEM containing 1% FBS. Then, cells were washed and cultured for 48 h. JEV-E protein in lysate and medium was detected by western blot analysis and quantified with Image Gauge ver. 3.1. JEV-E protein levels in lysate were normalized to actin levels. (**b**) After 48 h of JEV infection (MOI = 1), cells were fixed and stained with anti-JEV-E primary antibody and Alexa 564 anti-rabbit IgG secondary antibody. Nuclei were detected with Hoechst 33342, and images were obtained with confocal microscopy. Scale bars, 10 μm. (**c**) The plaque assay was performed using BHK cells. The medium was harvested from JEV-infected tMEFs culture after 48 h. Each medium was diluted with MEM-α and utilized for plaque assays. The numbers of plaques were counted and presented as forming units (PFU). (**d–j**) WT and SMS DKO tMEFs were pre-treated 20 mU/mL bacterial sphingomyelinase (BSM) for 10 min (**d**–**g**) and 5 μM C_6_-SM for 20 min (**h–j**), respectively. (**d** and **h**) SM was stained with MBP-conjugated lysenin. Bars, 10 μm. (**e** and **i**) Membrane SM was detected by Venus-lysenin and quantified as mean fluorescence intensity (MFI). (**f**) Cellular SM levels were assessed by LC-MS/MS. (**g** and **j**) After treatment with BSM or C_6_-SM, cells were infected with JEV (MOI = 0.1) for 1 h. After 48 h infection, JEV-E protein and actin were detected by immunoblotting and quantified. (**k**) JEV were pre-treated with 10 μM phosphatidylcholine liposome (PCL) or SM liposome (SML) for 1 h, and then utilized for infection to WT tMEFs (MOI = 1). After 48 h infection, JEV-E protein and actin were detected by immunoblotting and quantified. The values represent the mean ± SD (n = 3). *P < 0.005, **P < 0.05. Full-length blots are presented in Supplementary Figure S3.

To assess whether SM is associated with JEV infection, we infected tMEFs with JEV at various concentrations (from 0 to 1 MOI) for 1 h and detected the levels of JEV-E protein produced. At 48 h after infection, JEV-E protein production was elevated in a dose-dependent manner in WT tMEFs (Fig. 2a). JEV-E protein in both cell lysates and medium were reduced in SMS DKO tMEFs compared with WT tMEFs (Fig. 2a). Immunostaining with anti-JEV-E protein antibodies showed a significant decrease of JEV infection in SMS DKO tMEFs (Fig. 2b). Next, we performed viral plaque assay by using baby hamster kidney (BHK) cells and medium of tMEFs infected with JEV to assess JEV titer (Fig. 2c). According to reduction of JEV-E protein, the plaque forming units of SMS DKO tMEFs medium was lower than that of WT tMEFs. These results suggested that SMS deficiency leads to attenuation of JEV infection.

We further examined whether reduction of membrane SM blocks JEV infection. WT tMEFs were treated with bacterial sphingomyelinase (BSM), which is an enzyme for hydrolysis of SM to ceramide, to decrease SM on the plasma membrane. Indeed, BSM treatment decreased SM in lysenin staining (Fig. 2d and e) and LC-MS/MS (Fig. 2f and Supplementary Figure S1a). According to reduction of SM levels, BSM treatment also reduced JEV infection (Fig. 2g). Inversely, we tried recovery of SM by C_6_-SM treatment in SMS DKO tMEFs. Supplementation of C_6_-SM increased cell surface SM content in SMS DKO tMEFs (Fig. 2h and i). According to recovery of membrane SM, JEV-E protein was increased at 48 h after infection (Fig. 2j). These results suggested that JEV infection is related to membrane SM production by SMSs. To elucidate the relationship of SM with JEV infection, we performed pre-treatment of JEV with SM liposomes (SML) to absorb the virus. As shown in Fig. 2k, pre-treatment with SML significantly blocked JEV infection to WT tMEF compared to vehicle treatment. However, control PC liposomes (PCL) had no inhibitory effect on JEV infection (Fig. 2k). In addition, to clarify whether inhibition of virus infection by SM depletion is the specific effect for JEV or not, we performed infection of lentivirus containing GFP in WT and SMS DKO tMEFs. After 48 h, ratio of GFP positive cells were not decreased in SMS DKO tMEF compared with WT tMEF (Supplementary Figure S2). These results strongly suggested that JEV could specifically bind to SM, and SM was implicated in JEV infection.

### SMS deficiency inhibits JEV attachment and entry

**Figure 3:**
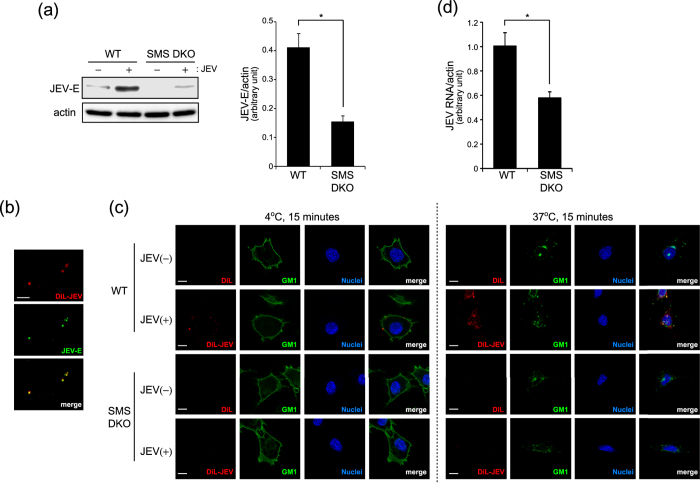
SMS deficiency inhibits JEV attachment. (**a**) To assess JEV attachment in tMEFs, medium of Vero cells infected with JEV (MOI = 1) for 48 h was used for short term (15 min) infection. Wild type (WT) and SMS knockout (SMS DKO) MEFs were infected with medium containing JEV for 15 min, washed, and harvested. JEV-E and actin were detected by western blot and quantified. The values represent the mean ± SD (n = 3). *P < 0.005. Full-length blots are presented in Supplementary Figure S4. (**b**) DiL-labelled JEV was added to poly-d-lysin-coated coverslip, stained with anti-JEV-E antibody and Alexa 488-conjugated rat IgG. Scale bars, 10 μm. (**c**) WT and SMS DKO MEFs were treated with DiL-labeled JEV and CT-b-AF488 for 15 min at 4 °C and 37 °C to detect the attachment and internalization of JEV. After fixation, nuclei were stained with Hoechst 33342. Scale bars, 10 μm. (**d**) To assess the uptake of JEV, virus entry assay was performed. Cells were infected with medium containing JEV for 1 h on ice, washed, and incubated for 15 min at 37 °C. After treatment with trypsin to remove cell surface virus, cells were harvested for real-time PCR. JEV RNA levels in the cells were normalized with β-actin mRNA. The values represent the mean ± SD (n = 3). *P < 0.005.

JEV infection is initiated after attachment of the virus to the cell surface of target cells through the E protein. Then JEV enters into the cell, replicates, assembles, and finally is released. From the above data, JEV-E production in the cell lysate and release into the culture media were suppressed in SM deficient cells. Thus we focused on the entry of JEV into target cells and examined JEV attachment shortly after infection in tMEFs. After 15 min infection with JEV, intracellular JEV-E protein was detected by western blot analysis. As shown in Fig. 3a, JEV-E protein was decreased in SMS DKO tMEFs compared with WT tMEFs. Next, we labelled JEV with DiL, which stains membrane lipids, to detect attachment of JEV to target cells. Notably, DiL-labelled JEV co-localized with JEV-E protein (Fig. 3b). To assess the effect of SM on JEV attachment and entry, we treated cells with DiL-JEV at 4 °C or 37 °C for 15 min. After infection with DiL-JEV for 15 min at 4 °C and 37 °C, both cell surface and intracellular DiL-JEV were detected in WT tMEFs (Fig. 3c). However, labelled JEV were undetectable in SMS DKO tMEFs. Previously, it was reported that JEV entry occurred via lipid microdomains (also known as lipid rafts)^21^,^22^. In addition, JEV invaded cells via GM1, a GSL localized in lipid microdomains the same as SM. At 4 °C, DiL-JEV was located on cell surface stained with GM1 by cholera toxin subunit B (CT-b). Then, DiL-JEV was partially colocalized with intracellular GM1 in WT tMEFs after 15 min incubation at 37 °C (Fig. 3c). To confirm that SM is related in uptake of JEV, we performed viral entry assay and quantitative real-time PCR analysis to detect intracellular JEV RNA. As shown Fig. 3d, amount of intracellular JEV RNA was significantly reduced in SMS DKO tMEFs compared with WT tMEFs. These data suggested that deficiency of SM prevents JEV attachment and entry through lipid microdomains.

### SMS1, but not SMS2, affects membrane SM levels for JEV attachment and infection

**Figure 4:**
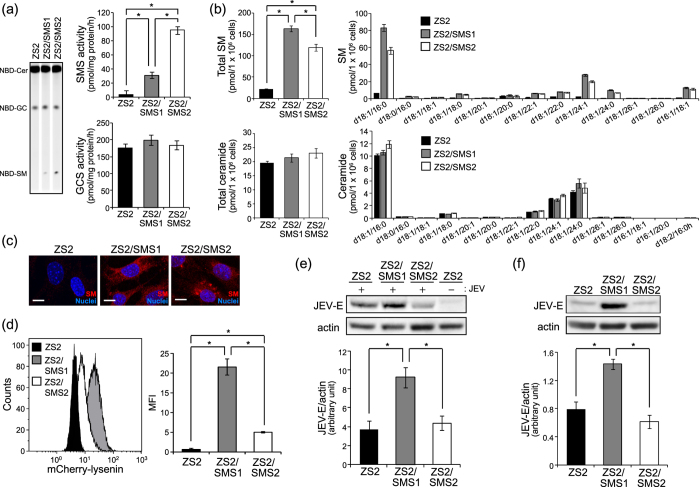
SMS1, but not SMS2, is required for infection and attachment of JEV. SMS DKO tMEFs stably expressing empty vector (ZS2), mouse SMS1 (ZS2/SMS1), or SMS2 (ZS2/SMS2) were prepared. (**a**) *In vitro* SMS and GCS activities were measured in lysate (100 μg protein) of cells using C_6_-NBD-ceramide as a substrate as described in the Materials and Methods. The values are the mean ± SD (n = 3). *P < 0.005. (**b**) Measurements of SM and ceramide were performed by LC-MS/MS. The value was mean ± SD (n = 3). *P < 0.005. (**c**) SM was detected with MBP-lysenin. Nuclei were stained with Hoechst 33342. Scale bars, 10 μm. (**d**) Membrane SM was detected with mCherry-lysenin and flowcytometer. Mean fluorescence intensity (MFI) was quantified with Kaluza software. (**e**) To assessed the JEV infection, cells were infected with JEV (MOI = 0.1) for 1 h, washed, and cultured for 48 h. JEV-E and actin were detected by immunoblotting and quantified. (**f**) For JEV attachment, cells were treated for 15 min with JEV-containing medium derived from Vero cells culture infected with JEV (MOI = 1) for 48 h. Then, JEV-E and actin were detected by western blot analysis and quantified. The presented values are the mean ± SD (n = 3). *P < 0.005. Full-length blots are presented in Supplementary Figure S5.

SMS has two isoforms, SMS1 and SMS2, which produce SM from ceramide and PC. SMS1 localizes to the Golgi apparatus, and SMS2 is in both the Golgi and plasma membrane. However, the relative contribution of SMS1 and SMS2 to membrane SM production for JEV attachment and infection is unknown. To clarify this, we utilized the revertant tMEFs expressing SMS1 or SMS2 (ZS2 tMEFs). Both SMS1 and SMS2 recovered SMS activity without affecting GCS activity (Fig. 4a). According to SMS activity, SM levels were also increased in both SMS1 and SMS2 (Fig. 4b). Interestingly, lysenin staining showed that SMS1 expression (ZS2/SMS1) increased both cell surface and intracellular SM levels (Fig. 4c and d). However, ZS2/SMS2-tMEFs exhibited intracellular SM staining with lysenin (Fig. 4c) and only slightly increased cell surface SM compared with control tMEFs (ZS2) (Fig. 4d).

To assess the effect of SMS expression on JEV infection, we treated ZS2-tMEFs with JEV and detected JEV-E protein at 48 h after infection. As shown Fig. 4e, ZS2/SMS1 showed an increase of JEV-E protein compared with ZS2 control cells. However, SMS2 introduction could not increase the JEV infection (Fig. 4e). Next, we elucidated the attachment of JEV to ZS2 cells. After 15 minutes of infection with JEV, only SMS1 increased the intracellular JEV-E protein, while SMS2 expression had no effect on JEV-E levels (Fig. 4f). These results suggested that SMS1, but not SMS2, contributes to the production of membrane SM for JEV attachment to cell surface and infection.

### JEV infection is suppressed in SMS1-deficient mice

**Figure 5:**
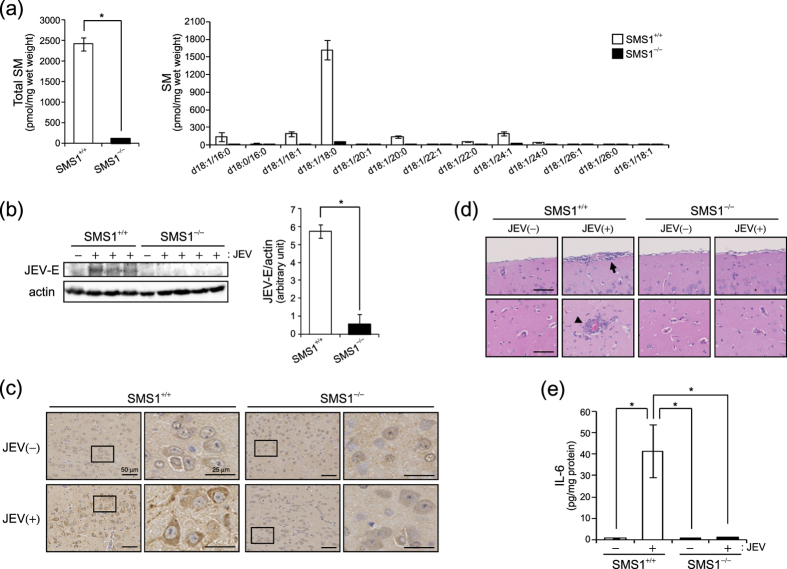
JEV infection of brains was decreased in SMS1 deficient mice. (**a**) SM levels were measured by LC-MS/MS in the brains of wild type (SMS1^+/+^) and SMS1 knockout (SMS1^−/−^) mice (n = 5). (**b–d**) SMS^+/+^ and SMS^−/−^ mice were intraperitoneally injected with JEV (1 × 10^4^ pfu/mouse) (n = 4) and were killed after 13 days. However, one JEV-injected SMS^+/+^ mouse was dead at 11 day. (**b**) JEV-E and actin were detected by western blot in brain lysates and quantified. The presented values are the mean ± SD (n = 3 [SMS1^+/+^] and 4 [SMS1^−/−^]). *P < 0.005. Full-length blots are presented in Supplementary Figure S6. (**c**) Immunohistochemistry using anti-JEV-E antibody and BCA staining in brain sections. (**d**) Haematoxylin and eosin staining of sections of brain. The arrow and arrow head indicate meningitis and leukocyte infiltration, respectively. Scale bars, 50 μm. (**e**) ELISA of IL-6 concentration in brain lysate. The values are the mean ± SD. *P < 0.005.

The above data showed that SMS1 but not SMS2 is important to generate membrane SM for JEV attachment and infection in tMEFs. Physiologically, JEV migrates to the brain and causes severe encephalitis. First, we elucidated the contribution of SMS1 to SM generation in the mouse brain. As shown Fig. 5a, SMS1-KO (SMS1^−/−^) significantly reduced almost all species of SM compared with WT (SMS1^+/+^) mice. In contrast, SMS2 deficient mice exhibited no change in SM content in the brain (data not shown). These data suggested that SMS1 contributes to the brain SM generation.

To investigate the physiological role of SM and SMS1 on JEV infection and toxicity, we performed JEV infection in WT (SMS1^+/+^) and SMS1^−/−^ mice. JEV (1 × 10^4^ PFU/mouse) was intraperitoneally injected into each of four mice per group and mice followed for 2 weeks. One SMS1^+/+^ mouse was dead on day 11 after JEV infection because of severe encephalitis. After 13 days, JEV infection in the brain was examined by western blot analysis and immunohistochemistry of JEV-E protein (Fig. 5b and c). JEV-E protein was detected in the brain of wild type SMS1^+/+^ mice (Fig. 5b). SMS1^−/−^ mice, however, did not have detectable levels of JEV-E protein (Fig. 5b). JEV-E protein was also detected in SMS1^+/+^ brains infected with JEV compared with vehicle-injected SMS1^+/+^ mice (Fig. 5c). Interestingly, JEV-E was not detected in SMS1^−/−^ mice injected with JEV (Fig. 5c). Next, we assessed pathological conditions in the brains of mice injected with or without JEV. JEV infection is well-known to induce inflammatory pathologies such as meningitis, leukocyte infiltration, and enhancement of inflammatory cytokines in the brain. Indeed, JEV infection in SMS1^+/+^ mice showed both meningitis (arrow) and leukocyte infiltration (arrow head) (Fig. 5d). However, we could not detect these pathologies in SMS1^−/−^ mice injected with JEV (Fig. 5d). Similarly, interleukin 6 (IL-6), a well-known inflammatory cytokine, was increased in JEV-infected SMS1^+/+^ mice (Fig. 5e). However, IL-6 levels were unchanged in SMS1^−/−^ mice after injection of JEV. These results suggest that SMS1 deficiency blocks JEV infection of the mouse brain.

## Discussion

In this study, we demonstrated that plasma membrane SM of target cells is implicated in the attachment and infection of JEV. First, we utilized SMS-deficient cells established from SMS1/SMS2 double knockout mice, and showed that JEV infection is attenuated by SMS deficiency at 48 h after treatment with JEV. This reduction of JEV infection was confirmed by SM depletion with bacterial SMase on WT tMEF and supplementation of SM in SMS DKO tMEF. Second, we examined the role of SM in the attachment of JEV to the surface of target cells. SM deficiency also suppressed binding of JEV to the cell surface and cell entry. These results suggested that JEV enters into target cells through plasma membrane SM. Then, we elucidate which of SMS1 or SMS2 contributes to SM production necessary for JEV attachment and entry. Although overexpression of both SMS1 and SMS2 in SMS DKO tMEFs recovered cellular SM levels, only SMS1 could produce plasma membrane SM. According to plasma membrane SM levels, JEV attachment and infection were elevated in SMS1-expressing tMEFs. Finally, we performed a JEV infection model in SMS1-deficient mice. SMS1-deficient mice exhibited resistance to JEV infection of the brain. These results suggested that SMS1-generated SM on the plasma membrane is essential for JEV attachment and infection.

In the present study, we elucidated JEV levels by western blot analysis of JEV-E protein in cell lysate and medium. Extracellular E protein in the medium is correlated with released JEV levels. In contrast, intracellular JEV in the cell lysate correlates with replicating JEV levels. Taffese *et al*. showed that SMS1 deficiency in diploid cells decreases influenza virus production but not entry^28^. Influenza virus utilizes the same pathway of protein release when releasing virus particles. Thus, intracellular virus amounts were unchanged in SMS1 deficient cells compared with wild type cells. However, our results showed both lysate and medium JEV-E proteins were reduced in SMS1/2 double knockout tMEFs (SMS DKO tMEFs), suggesting that JEV production is also decreased, and SM is associated with attachment and entry of JEV.

Recently, Tani *et al*. reported that SM hydrolysis by treatment with BSM enhances JEV infection in Huh7 cells, suggesting that ceramide production from SM by sphingomyelinase (SMase) is implicated in JEV entry^29^. However, in our data, BSM treatment inversely decreased JEV infection with SM depletion in plasma membrane. In addition, we tested the relationship of viral infection with endogenous SMase. There are two types of SMase related to cellular SM hydrolysis, neutral SMase and acid SMase which regulate various cellular functions such as proliferation, apoptosis, autophagy, and virus infection^30^,^31^. However, inhibitors of both neutral SMase (GW 4869) and acid SMase (desipramine) had no effect on JEV infection (data not shown). Their treatment time is longer (1 h) than our condition (10 min). Previously, we tried the BSM treatment^25^ and found that 10 min treatment is sufficient to deplete membrane SM (Supplementary Figure S1a). Moreover, in our condition, ceramide levels were markedly increased by BSM treatment (Supplementary Figure S1b). Inversely, BSM had no effect on SM and ceramide in SMS DKO tMEFs (Supplementary Figure S1a and b). These data suggested that BSM really converts SM to ceramide and reduced JEV infection. However, the opposition of data in JEV infection on BSM treatment remains unclear. Perhaps, longer treatment might have different effects on JEV attachment or entry through SM metabolism. Alternatively, infection mechanisms may be different between hepatoma Huh7 cells and MEFs, as JEV is known to have numerous entry pathways such as clathrin, caveolae, and lipid raft in various cell types.

SM is found in clathrin-coated pits, caveolae, and lipid rafts. Previously, we showed that SM regulates clathrin-dependent endocytosis of transferrin and its receptor in mouse leukocyte WR19L cells^25^, and inhibits excess response to CXCL12 in lipid rafts in WT and SMS DKO tMEFs^27^. JEV has multiple entry processes: clathrin-dependent endocytosis in mosquito cells^14^ and the porcine kidney epithelial PK15 cells^18^; the clathrin-independent pathway in mouse and human neuronal cell lines^19^; caveola-medicated entry in rat neuroblastoma cells^20^; lipid raft in hepatoma Huh7 cells^21^ and neural stem/progenitor cells^22^. SM and SMS can be associated with all processes to regulate JEV attachment and entry because of its localization. However, SM depletion in SMS DKO tMEFs could not completely inhibit JEV infection, which suggested the existence of a SM-independent pathway or small remaining amounts of SM in SMS DKO tMEFs. In addition, it is unknown whether SM regulates JEV attachment independently or with other receptor candidates such as Hsp70. To elucidate its molecular mechanisms are our further investigation.

In an animal model of JEV infection, JEV-infected mice exhibit multiple pathologies such as meningitis, leukocyte infiltration, and elevation of inflammatory cytokines^32^,^33^. Our data confirm these pathologies in WT mice after injection with JEV. However, we could not detect these phenomena in SMS1^−/−^ mice brains. Indeed, although one mouse was dead on day 11 and 2 mice had decreased body weight at 13 days in WT mice (data not shown), all SMS1^−/−^ mice survived until 13 days and did not have severe weight loss. These results suggest that SMS1 is implicated in JEV infection *in vivo*, as well as *in vitro*.

In conclusion, our present work demonstrated SMS1/SM-dependent JEV infection in cells and mice. SM-depleted cells reduced JEV attachment and entry into target cells. SMS1, but not SMS2, contributed to SM generation inducing JEV attachment and infection. Our findings suggest that SMS1 and SM may be a target to inhibit JEV infection.

## Materials and Methods

### Materials

Materials were purchased as follows: C_6_-NBD ceramide and C_6_-sphingomyelin (Matreya, Pleasant Gap, PA, USA); bacterial sphingomyelinase (BSM) and Hoechst33342 (Sigma-Aldrich, St. Louis, MO, USA); anti-β-actin antibodies (Santa Cruz Biotechnology Inc., Dallas, TX, USA); horseradish peroxidase-conjugated secondary antibodies (Promega, Madison, WI, USA); Alexa Fluor 488-conjugated cholera toxin subunit B (CT-b), Alexa-conjugated secondary antibodies and DiL cell-labelling solution (Molecular Probes, Eugene, OR, USA); anti-maltose binding protein (anti-MBP) monoclonal antibody (Upstate Biotechnology, Waltham, MA, USA). Rabbit anti-JEV-E antibody was made previously^34^,^35^,^36^. MBP-conjugated, mCherry-conjugated, and Venus-conjugated lysenin were kindly gifted by Dr Kobayashi (RIKEN, Saitama, Japan). Phosphatidylcholine (PC) from milk and SM from milk were from Nagara Science (Gifu, Japan). To make the PC liposomes and SM liposomes, PC and SM were solved in distilled water at 10 mM concentration and sonicated until becoming clear solution.

### Cell culture and virus infection

The JEV JaGAr-01 strain was employed for this study. For virus production, the African green monkey kidney cell line Vero was infected with JEV for 1 h at 10 multiplicity of infection (MOI) per cell. After removing virus solution, cells were cultured in MEM containing 5% (v/v) foetal bovine serum (FBS) for 24 h. Viruses in medium were titered by the plaque method using baby hamster kidney (BHK) cells as described previously^37^,^38^. Mouse embryonic fibroblasts (tMEF) immortalized by the SV40 large T antigen were established from wild type (WT) and SMS1/2 double knockout (SMS DKO) mice previously^26^,^27^. tMEFs were cultured in DMEM supplemented with 10% (v/v) FBS, 100 units/mL penicillin, and 100 μg/mL streptomycin. For JEV infection (24 and 48 h), tMEFs were cultured in DMEM containing 1% (v/v) FBS and treated with JEV at various MOI for 1 h. After removing virus, cells were cultured in DMEM with 10% (v/v) FBS. For JEV attachment (15 min infection), high amounts of JEV were produced from 5 mL culture medium of BHK cells infected with 1 MOI for 48 h. Then, tMEFs were treated with 500 μL of JEV solution for 15 min and used for assays.

For DiL-labelled virus^19^,^39^,^40^, JEV was labelled with 400 nM DiL for 10 min at room temperature. Excess dye was removed by purification through micro spin G-25 column (GE Healthcare Life Sciences, Little Chalfont, UK). tMEFs were treated with DiL-labelled JEV and CT-b-AF488 for 15 min at 4 °C or at 37 °C. Then DiL-JEV and CT-b-AF488 were detected by immunocytochemistry.

### Sphingomyelin synthase and glucosylceramide synthase activities

Sphingomyelin synthase (SMS) and glucosylceramide synthase (GCS) activities were determined as described previously^41^. Briefly, cells were homogenized in ice-cold buffer (20 mM Tris-HCl, pH 7.4, 2 mM EDTA, 10 mM EGTA, 1 mM phenylmethylsulfonyl fluoride (PMSF), 2.5 μg/ml leupeptin, and 2.5 μg/ml aprotinin). Total protein (100 μg) was added to the reaction solution (10 mM Tris-HCl, pH 7.5, 1 mM EDTA, 20 μM C_6_-NBD-ceramide, 120 μM phosphatidylcholine [PC], 0.1 mM UDP-glucose) and incubated for 60 min at 37 °C. Lipids were extracted and separated by thin-layer chromatography on silica gel plates using the solvent containing chloroform-methanol-12 mM MgCl_2_ (65:25:4, v/v/v). Fluorescent lipids were detected by the LAS-4000 system (Fujifilm, Tokyo, Japan) and quantified with Image Gauge 3.1 software (Fujifilm).

### Immunocytochemistry and flow cytometry

Membrane SM was detected by lysenin conjugated MBP or mCherry. For detection of JEV-E protein and SM by immunocytochemistry, cells were cultured on poly-L-lysine-coated chamber slides. After each treatment, cells were washed with ice-cold PBS and then fixed with 1% (w/v) paraformaldehyde (PFA) for 10 min at 4 °C. After washing with ice-cold PBS, cells were treated with MBP-conjugated lysenin for 30 min at 4 °C and fixed with 2% (v/v) PFA at 4 °C. Then, cells were permeabilized with PBS containing 0.2% (v/v) Triton X-100 for 5 min and incubated with PBS containing 2% (w/v) BSA for 30 min at room temperature. Cells were washed with PBS and incubated with primary antibodies against MBP and JEV-E proteins for 90 min at room temperature. After washing with PBS, AF488- or AF546-conjugated anti-IgG antibodies were incubated for 45 min. Nuclei were counterstained with Hoechst33342. Specimens were observed with the LSM710 confocal microscopy (Carl Zeiss, Jene, Germany) with 63× objective lens and analysed with ZEN software.

To detect membrane SM levels by flow cytometry, cells were detached with 0.25% (w/v) trypsin/EDTA solution and fixed with 0.3% (w/v) glutaraldehyde/2% (w/v) PFA for 10 min at 4 °C. After washing with PBS, cells were stained with mCherry-conjugated or Venus-conjugated lysenin for 30 min at room temperature. Cells were analysed with a Gallios flow cytometer (Beckman Coulter, Miami, FL, USA). Fluorescence were quantified with Kaluza software (Beckman Coulter) and represented as mean fluorescence intensity (MFI).

### Liquid chromatography tandem mass spectrometry (LC-MS/MS)

Lipid extraction from tMEFs or mouse brain and measurements of ceramide species in lipid extracts were performed using LC-MS/MS as described previously^42^,^43^. The amounts of SM and ceramide species with various carbon chains (d18:1/16:0, d18:0/16:0, d18:1/18:1, d18:1/18:0, d18:1/20:1, d18:1/20:0, d18:1/22:1, d18:1/22:0, d18:1/24:1, d18:1/24:0, d18:1/26:1, d18:1/26:0, d16:1/18:1, d16:1/20:0, and d18:2/16:0 h) were determined. Each sphingolipid was normalized with cell numbers or PC amount.

### Western blot analysis

To detect JEV-E protein by western blot analysis, cells or media were harvested. Cells were lysed in lysis buffer (10 mM Tris-HCl, pH 7.4, 10 mM KCl, 1.5 mM MgCl_2_, 1% (v/v) Triton X-100, 1 mM PMSF, 10 μg/ml leupeptin and 10 μg/ml aprotinin). After incubation on ice for 20 min, debris was removed by centrifugation at 2,000 × *g* for 10 min at 4 °C. Supernatant and media were used as a loading sample. Proteins (30 μg) were subjected to SDS-poly-acrylamide gel electrophoresis and transferred to polyvinylidene difluoride membranes (Millipore, Bedford, MA, USA). Nonspecific binding was blocked by incubation of the membrane with PBS containing 0.1% (v/v) Tween-20 (PBS-T) and 5% (w/v) non-fat dried milk for 20 min at room temperature. Then membrane was incubated with primary antibodies overnight at 4 °C and with secondary antibodies for 45 min at room temperature. Immunoreactive protein bands were visualized using an ECL-peroxidase detection system (Amersham Biosciences, Piscataway, NJ, USA) and LAS-4000 (Fujifilm, Tokyo, Japan), and calculated with Image Gauge. JEV infection levels indicated as the ratio of JEV-E to actin.

### Plaque assay

The plaque assay was performed as descried previously^37^,^44^. BHK cells were seeded in 24-well plates at a concentration of 1.8 × 10^5^ cells. After 24 h, cells were incubated with 150 μL of diluted medium with MEM-α for 1 h, washed, and incubated in E-MEM supplemented with 1.25% (w/v) methylcellulose, 2% (v/v) FBS, and 2 mM glutamine. After 3 days of infection, cells were fixed with cold-methanol and stained with 1% (v/v) crystal violet solution.

### Viral entry assay and quantitative real-time PCR

WT and SMS DKO tMEFs were infected with JEV for 1 h on ice. After washing unbound JEV, cells were incubated for 15 min at 37 °C. Then, cells were treated with 0.05% (w/v) trypsin for 5 min at 37 °C to remove JEV bound to cell surface. After inactivation of trypsin with DMEM containing 10% FBS, cells were washed and harvested. Total RNAs were extracted using an RNA purification kit (RNeasy, Qiagen, Hilden, Germany), and 1 μg of total RNA was converted to complementary DNA (cDNA) using ReverTra Ace qPCR RT Kit (Toyobo, Osaka, Japan). Quantitative real-time PCR was performed using 12 K Flex Real-Time PCR System (ThermoFisher Scientific, Rockford, IL, USA), according to the standard TaqMan PCR kit protocol. The following primer and probe sets were used for JEV RNA quantification: forward primer 5′CTCAGCCTCTAAACGCCTATCC-3′, reverse primer 5′-GTCTCAGGTCCATCTACGACAAATG-3′, and probe 5′-AGAGAGCATTCTTTTTGCCCCGGAATTG-3′. The mixture of the primers and the probe for β-actin as an internal reference control was purchased from Thermo Fisher Scientific (TaqMan Gene Expression assay, Mm00607939_s1, Vic, Primer Limited). JEV RNA levels were normalized by β-actin mRNA.

### JEV infection and viral pathogenicity in mice

*Sgms1*-deficient (SMS1^−/−^) mice were generated using D3 embryonic stem cells followed by backcrossing to the C57BL/6 strain, as previously reported^45^. Wild type C57BL/6 J (SMS1^+/+^) mice were purchased from Clea Japan (Tokyo, Japan). Mice were housed and maintained under specific pathogen-free conditions at the animal centre of Kanazawa Medical University. All experiments were approved by the Committee for Animal Experiments of Kanazawa Medical University, and performed in accordance with the Guidelines for Animal Experimentation of Kanazawa Medical University. Four mice aged 4–5 weeks of age were intraperitoneally injected with JEV at 1 × 10^4^ plaque-forming units (PFU) and followed. After 13 days, mice brains were prepared and used for western blot analysis, immunohistochemistry, and ELISA.

### Immunohistochemistry

The mouse brains were fixed with 4% (w/v) PFA and embedded in paraffin. Brain sections (3–5 μm) were cut and prepared for immunostaining by deparaffinising in xylene, passing through 100% (v/v), 98% (v/v), and 80% (v/v) ethanol, and washing with PBS. Antigen retrieval was performed by heating in Tris-EDTA (pH 9.0) buffer. Endogenous peroxidases were inactivated with 3% (v/v) H_2_O_2_ in methanol for 10 min at room temperature. Sections were blocked with 10% (v/v) normal rabbit serum and treated with rabbit anti-JEV-E antibody overnight at room temperature. The peroxidase labelling was performed with 3.3′-diaminobenzidine (DAB) staining for 5 min and counterstaining with Mayer’s haematoxylin. Then, slides were mounted and analysed using a NanoZoomer slide scanner (Hamamatsu Photonics, Shizuoka, Japan).

### Enzyme-linked immunosorbent assay (ELISA)

Murine interleukin 6 (IL-6) was measured using a commercial ELISA kit (ADI-900-045, Enzo Life Sciences, Ann Arbor, MI, USA) according to the manufacturer’s protocol. Mouse brains were lysed in lysis buffer and homogenized. After incubation on ice for 20 min, debris was removed by centrifugation at 2,000 × *g* for 10 min at 4 °C. Supernatants were used for both ELISA and western blot analysis.

### Statistical Analysis

The results are expressed as the mean ± standard deviation (SD). Statistical comparisons between experimental groups were carried out using the unpaired Student’s *t*-test. Differences between groups with *P*-values of *p* < 0.05 were considered statistically significant.

## Additional Information

**How to cite this article**: Taniguchi, M. *et al*. Sphingomyelin generated by sphingomyelin synthase 1 is involved in attachment and infection with Japanese encephalitis virus. *Sci. Rep.* **6**, 37829; doi: 10.1038/srep37829 (2016).

**Publisher's note:** Springer Nature remains neutral with regard to jurisdictional claims in published maps and institutional affiliations.

## Supplementary Material

Supplementary Information

## References

[b1] Hannun Y. A. & Obeid L. M. Principles of bioactive lipid signalling: lessons from sphingolipids. Nat Rev Mol Cell Biol 9, 139–150 (2008). 18216770 10.1038/nrm2329

[b2] Schneider-Schaulies J. & Schneider-Schaulies S. Viral infections and sphingolipids. Handb Exp Pharmacol 321–340 (2013). 23563664 10.1007/978-3-7091-1511-4_16

[b3] Schneider-Schaulies J. & Schneider-Schaulies S. Sphingolipids in viral infection. Biol Chem 396, 585–595 (2015). 25525752 10.1515/hsz-2014-0273

[b4] Puri A. . An inhibitor of glycosphingolipid metabolism blocks HIV-1 infection of primary T-cells. AIDS 18, 849–858 (2004). 15060432 10.1097/00002030-200404090-00002

[b5] Rawat S. S. . Elevated expression of GM3 in receptor-bearing targets confers resistance to human immunodeficiency virus type 1 fusion. J Virol 78, 7360–7368 (2004). 15220409 10.1128/JVI.78.14.7360-7368.2004 PMC434090

[b6] Lund N. . A novel soluble mimic of the glycolipid, globotriaosyl ceramide inhibits HIV infection. AIDS 20, 333–343 (2006). 16439866 10.1097/01.aids.0000206499.78664.58

[b7] Lingwood C. A. . New aspects of the regulation of glycosphingolipid receptor function. Chem Phys Lipids 163, 27–35 (2010). 19781539 10.1016/j.chemphyslip.2009.09.001

[b8] Burckhardt C. J. & Greber U. F. Virus movements on the plasma membrane support infection and transmission between cells. PLoS Pathog 5, e1000621 (2009). 19956678 10.1371/journal.ppat.1000621 PMC2777510

[b9] Dreschers S. . Infections with human rhinovirus induce the formation of distinct functional membrane domains. Cell Physiol Biochem 20, 241–254 (2007). 17595532 10.1159/000104170

[b10] Grassme H., Riehle A., Wilker B. & Gulbins E. Rhinoviruses infect human epithelial cells via ceramide-enriched membrane platforms. J Biol Chem 280, 26256–26262 (2005). 15888438 10.1074/jbc.M500835200

[b11] Miller M. E., Adhikary S., Kolokoltsov A. A. & Davey R. A. Ebolavirus requires acid sphingomyelinase activity and plasma membrane sphingomyelin for infection. J Virol 86, 7473–7483 (2012). 22573858 10.1128/JVI.00136-12 PMC3416309

[b12] Gubler D., Kuno G. & Markoff L. Flaviviruses. (eds Knipe D. M. & Howley P. M.) *Fields virology*, 5th ed. Vol. 1. Lipincott-Williams & Wilkins, Philadelphia, PA. pp. 1153–1253 (2007).

[b13] Ren J., Ding T., Zhang W., Song J. & Ma W. Does Japanese encephalitis virus share the same cellular receptor with other mosquito-borne flaviviruses on the C6/36 mosquito cells? Virol J 4, 83 (2007). 17803826 10.1186/1743-422X-4-83 PMC2075493

[b14] Chuang C. K., Yang T. H., Chen T. H., Yang C. F. & Chen W. J. Heat shock cognate protein 70 isoform D is required for clathrin-dependent endocytosis of Japanese encephalitis virus in C6/36 cells. J Gen Virol 96, 793–803 (2015). 25502019 10.1099/jgv.0.000015

[b15] Lee E. & Lobigs M. Mechanism of virulence attenuation of glycosaminoglycan-binding variants of Japanese encephalitis virus and Murray Valley encephalitis virus. J Virol 76, 4901–4911 (2002). 11967307 10.1128/JVI.76.10.4901-4911.2002 PMC136177

[b16] Takada A. . A system for functional analysis of Ebola virus glycoprotein. Proc Natl Acad Sci USA 94, 14764–14769 (1997). 9405687 10.1073/pnas.94.26.14764 PMC25111

[b17] Boonsanay V. & Smith D. R. Entry into and production of the Japanese encephalitis virus from C6/36 cells. Intervirology 50, 85–92 (2007). 17139184 10.1159/000097394

[b18] Yang S. . Japanese encephalitis virus infects porcine kidney epithelial PK15 cells via clathrin- and cholesterol-dependent endocytosis. Virol J 10, 258 (2013). 23937769 10.1186/1743-422X-10-258 PMC3751042

[b19] Kalia M., Khasa R., Sharma M., Nain M. & Vrati S. Japanese encephalitis virus infects neuronal cells through a clathrin-independent endocytic mechanism. J Virol 87, 148–162 (2013). 23055570 10.1128/JVI.01399-12 PMC3536362

[b20] Zhu Y. Z. . Japanese encephalitis virus enters rat neuroblastoma cells via a pH-dependent, dynamin and caveola-mediated endocytosis pathway. J Virol 86, 13407–13422 (2012). 23015720 10.1128/JVI.00903-12 PMC3503063

[b21] Zhu Y. Z. . Association of heat-shock protein 70 with lipid rafts is required for Japanese encephalitis virus infection in Huh7 cells. J Gen Virol 93, 61–71 (2012). 21940409 10.1099/vir.0.034637-0

[b22] Das S., Chakraborty S. & Basu A. Critical role of lipid rafts in virus entry and activation of phosphoinositide 3′ kinase/Akt signaling during early stages of Japanese encephalitis virus infection in neural stem/progenitor cells. J Neurochem 115, 537–549 (2010). 20722967 10.1111/j.1471-4159.2010.06951.x

[b23] Taniguchi M. & Okazaki T. The role of sphingomyelin and sphingomyelin synthases in cell death, proliferation and migration-from cell and animal models to human disorders. Biochim Biophys Acta 1841, 692–703 (2014). 24355909 10.1016/j.bbalip.2013.12.003

[b24] Tafesse F. G., Ternes P. & Holthuis J. C. The multigenic sphingomyelin synthase family. J Biol Chem 281, 29421–29425 (2006). 16905542 10.1074/jbc.R600021200

[b25] Shakor A. B. . Sphingomyelin synthase 1-generated sphingomyelin plays an important role in transferrin trafficking and cell proliferation. J Biol Chem 286, 36053–36062 (2011). 21856749 10.1074/jbc.M111.228593 PMC3195583

[b26] Mitsutake S. . Dynamic modification of sphingomyelin in lipid microdomains controls development of obesity, fatty liver, and type 2 diabetes. J Biol Chem 286, 28544–28555 (2011). 21669879 10.1074/jbc.M111.255646 PMC3151096

[b27] Asano S. . Regulation of cell migration by sphingomyelin synthases: sphingomyelin in lipid rafts decreases responsiveness to signaling by the CXCL12/CXCR4 pathway. Mol Cell Biol 32, 3242–3252 (2012). 22688512 10.1128/MCB.00121-12 PMC3434554

[b28] Tafesse F. G. . Intact sphingomyelin biosynthetic pathway is essential for intracellular transport of influenza virus glycoproteins. Proc Natl Acad Sci USA 110, 6406–6411 (2013). 23576732 10.1073/pnas.1219909110 PMC3631694

[b29] Tani H. . Involvement of ceramide in the propagation of Japanese encephalitis virus. J Virol 84, 2798–2807 (2010). 20053738 10.1128/JVI.02499-09 PMC2826033

[b30] Kitatani K., Taniguchi M. & Okazaki T. Role of Sphingolipids and Metabolizing Enzymes in Hematological Malignancies. Mol Cells 38, 482–495 (2015). 25997737 10.14348/molcells.2015.0118 PMC4469906

[b31] Bienias K., Fiedorowicz A., Sadowska A., Prokopiuk S. & Car H. Regulation of sphingomyelin metabolism. Pharmacol Rep 68, 570–581 (2016). 26940196 10.1016/j.pharep.2015.12.008

[b32] Hayasaka D. . TNF-alpha acts as an immunoregulator in the mouse brain by reducing the incidence of severe disease following Japanese encephalitis virus infection. PLoS One 8, e71643 (2013). 23940775 10.1371/journal.pone.0071643 PMC3733918

[b33] Ghoshal A. . Proinflammatory mediators released by activated microglia induces neuronal death in Japanese encephalitis. Glia 55, 483–496 (2007). 17203475 10.1002/glia.20474

[b34] Takegami T., Miyamoto H., Nakamura H. & Yasui K. Differences in biological activity of the V3 envelope protein of two Japanese encephalitis virus strains. Acta Virol 26, 321–327 (1982). 6128901

[b35] Takegami T., Miyamoto H., Nakamura H. & Yasui K. Biological activities of the structural proteins of Japanese encephalitis virus. Acta Virol 26, 312–320 (1982). 6128900

[b36] Edward Z. & Takegami T. Localization and functions of Japanese encephalitis virus nonstructural proteins NS3 and NS5 for viral RNA synthesis in the infected cells. Microbiol Immunol 37, 239–243 (1993). 8321152 10.1111/j.1348-0421.1993.tb03206.x

[b37] Takegami T. & Hotta S. Synthesis and localization of Japanese encephalitis virus RNAs in the infected cells. Microbiol Immunol 34, 849–857 (1990). 1963921 10.1111/j.1348-0421.1990.tb01063.x

[b38] Murakami M., Ota T., Nukuzuma S. & Takegami T. Inhibitory effect of RNAi on Japanese encephalitis virus replication *in vitro* and *in vivo*. Microbiol Immunol 49, 1047–1056 (2005). 16365529 10.1111/j.1348-0421.2005.tb03701.x

[b39] Mosso C., Galvan-Mendoza I. J., Ludert J. E. & del Angel R. M. Endocytic pathway followed by dengue virus to infect the mosquito cell line C6/36 HT. Virology 378, 193–199 (2008). 18571214 10.1016/j.virol.2008.05.012

[b40] Ueda R. . A novel single virus infection system reveals that influenza virus preferentially infects cells in g1 phase. PLoS One 8, e67011 (2013). 23874406 10.1371/journal.pone.0067011 PMC3715512

[b41] Yamaoka S., Miyaji M., Kitano T., Umehara H. & Okazaki T. Expression cloning of a human cDNA restoring sphingomyelin synthesis and cell growth in sphingomyelin synthase-defective lymphoid cells. J Biol Chem 279, 18688–18693 (2004). 14976195 10.1074/jbc.M401205200

[b42] Ogiso H. . Comparative Analysis of Biological Sphingolipids with Glycerophospholipids and Diacylglycerol by LC-MS/MS. Metabolites 4, 98–114 (2014). 24958389 10.3390/metabo4010098 PMC4018675

[b43] Ogiso H., Taniguchi M. & Okazaki T. Analysis of lipid-composition changes in plasma membrane microdomains. J Lipid Res 56, 1594–1605 (2015). 26116739 10.1194/jlr.M059972 PMC4514000

[b44] Tasaki T., Nukuzuma S. & Takegami T. Impaired Japanese encephalitis virus replication in p62/SQSTM1 deficient mouse embryonic fibroblasts. Microbiol Immunol 60, 708–711 (2016). 27624873 10.1111/1348-0421.12440

[b45] Yano M. . Mitochondrial dysfunction and increased reactive oxygen species impair insulin secretion in sphingomyelin synthase 1-null mice. J Biol Chem 286, 3992–4002 (2011). 21115496 10.1074/jbc.M110.179176 PMC3030399

