# Supplementary material for: Sphingomyelin generated by sphingomyelin synthase 1 is involved in attachment and infection with Japanese encephalitis virus
Source: Sci Rep. 2016 Nov 28;6:37829. doi: 10.1038/srep37829 (PMC5124946; doi:10.1038/srep37829)
Supplement: Supplementary Information [file srep37829-s1.pdf]

Supplementary information

Sphingomyelin generated by sphingomyelin synthase 1 is involved in attachment and infection with Japanese encephalitis virus

Makoto Taniguchi[1]*, Takafumi Tasaki[1], Hideaki Ninomiya[2], Yoshibumi Ueda[3,4], Koh-ichi Kuremoto[5], Susumu Mitsutake[6], Yasuyuki Igarashi[7], Toshiro Okazaki[3], and Tsutomu Takegami[1]

[1] Department of Life Science, Medical Research Institute, [2] Histology Laboratory, Research Support Center, Medical Research Institute, and [3] Department of Hematology and Immunology, Kanazawa Medical University, 1-1 Daigaku, Uchinada, Ishikawa 920-0293, Japan

[4] Graduate School of Arts and Sciences, University of Tokyo, Komaba, Meguro-ku, Tokyo, 153-8902, Japan

[5] Department of Advanced Prosthodontics, Graduate School of Biomedical & Health Sciences, Hiroshima University, Hiroshima 734-8553, Japan

[6] Department of Applied Biochemistry and Food Sciences, Faculty of Agriculture, Saga University, Saga 840-8502, Japan

[7] Laboratory of Biomembrane and Biofunction Chemistry, Faculty of Advanced Life Sciences, Hokkaido University, Kita 21-jo, Nishi 11-chome, Kita-ku, Sapporo 001-0021, Japan

Supplementary Materials and Methods

Lentivirus infection. Lentivirus particles containing GFP were purchased from Sant Cruz Biothecnology (copGFP Control Lentiviral Particles, sc-108084). WT and SMS DKO tMEFs were seeded on 6 well plate and pre-treated with 1 μg/mL polybrene (Santa Crus Biotechnology) for 20 min at 37°C. Cells were infected for 1 h at 1 MOI, washed with PBS, and cultured. After 48 h infection, GFP-positive cells were observed with fluorescent microscopy BZ-9000 (KEYENCE, Osaka, Japan) and analysed with Gallios (Beckman).

Supplementary Figure Legends

Supplementary Figure S1. Measurement of SM and ceramide after treatment with BSM in WT and SMS DKO tMEFs.

WT and SMS DKO tMEFs were treated with 20 mU/mL BSM for 10 min and then harvested after washing. SM (**a**) and ceramide (**b**) levels were assessed by LC-MS/MS and normalized with total PC amounts. The value presented is the mean ± SD (n = 3). * $P < 0.005$.

Supplementary Figure S2. Lentivirus infection in WT and SMS DKO tMEFs.

WT and SMS DKO tMEFs were infected for 1 h at 1 MOI. After removing unbound lentivirus, cells were cultured for 48 h. (**a**) Cells were observed with fluorescent microscopy, Scale bars, 20 μm. (**b**) Flowcytometry analysis of GFP-positive cells. The value presented is the mean ± SD (n = 3). * $P < 0.005$.

Supplementary Figure S3.

(a) Full-length blots of Figure 2(a). (b) Full-length blots of Figure 2(g). (c) Full-length blots of Figure 2(j). (d) Full-length blots of Figure 2(k).

Supplementary Figure S4.

Full-length blots for main figures corresponded to Figure 3(a).

Supplementary Figure S5.

(a) Full-length blots of Figure 4(e). (b) Full-length blots of Figure 4(f).

Supplementary Figure S6.

Full-length blots for main figures corresponded to Figure 5(b).

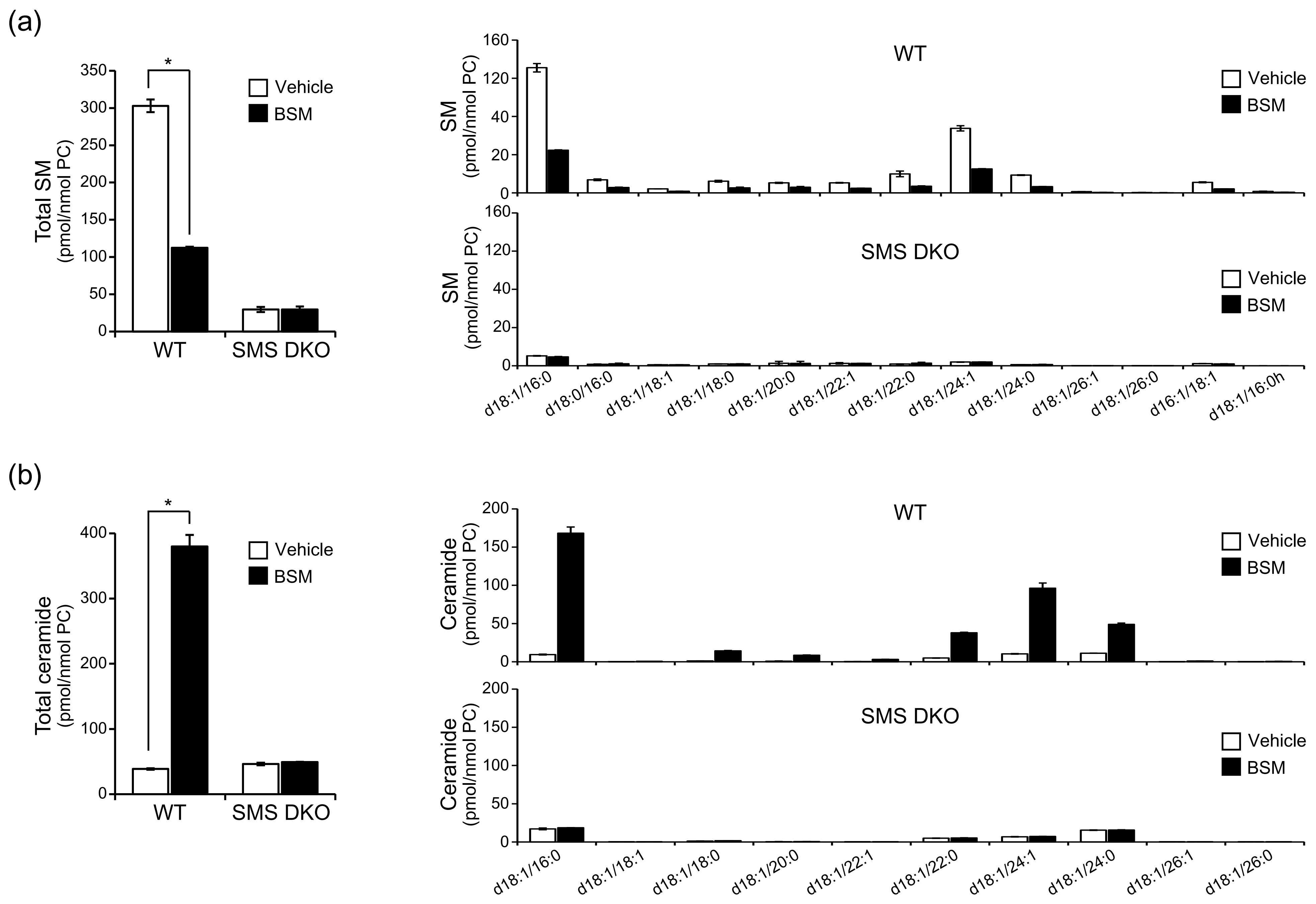
(a)
Total SM (pmol/nmol PC)
Vehicle
BSM
WT
SMS DKO
SM (pmol/nmol PC)
d18:1/16:0
d18:0/16:0
d18:1/18:1
d18:1/18:0
d18:1/20:0
d18:1/22:1
d18:1/22:0
d18:1/24:1
d18:1/24:0
d18:1/26:1
d18:1/26:0
d16:1/18:1
d18:1/16:0h
(b)
Total ceramide (pmol/nmol PC)
Ceramide (pmol/nmol PC)

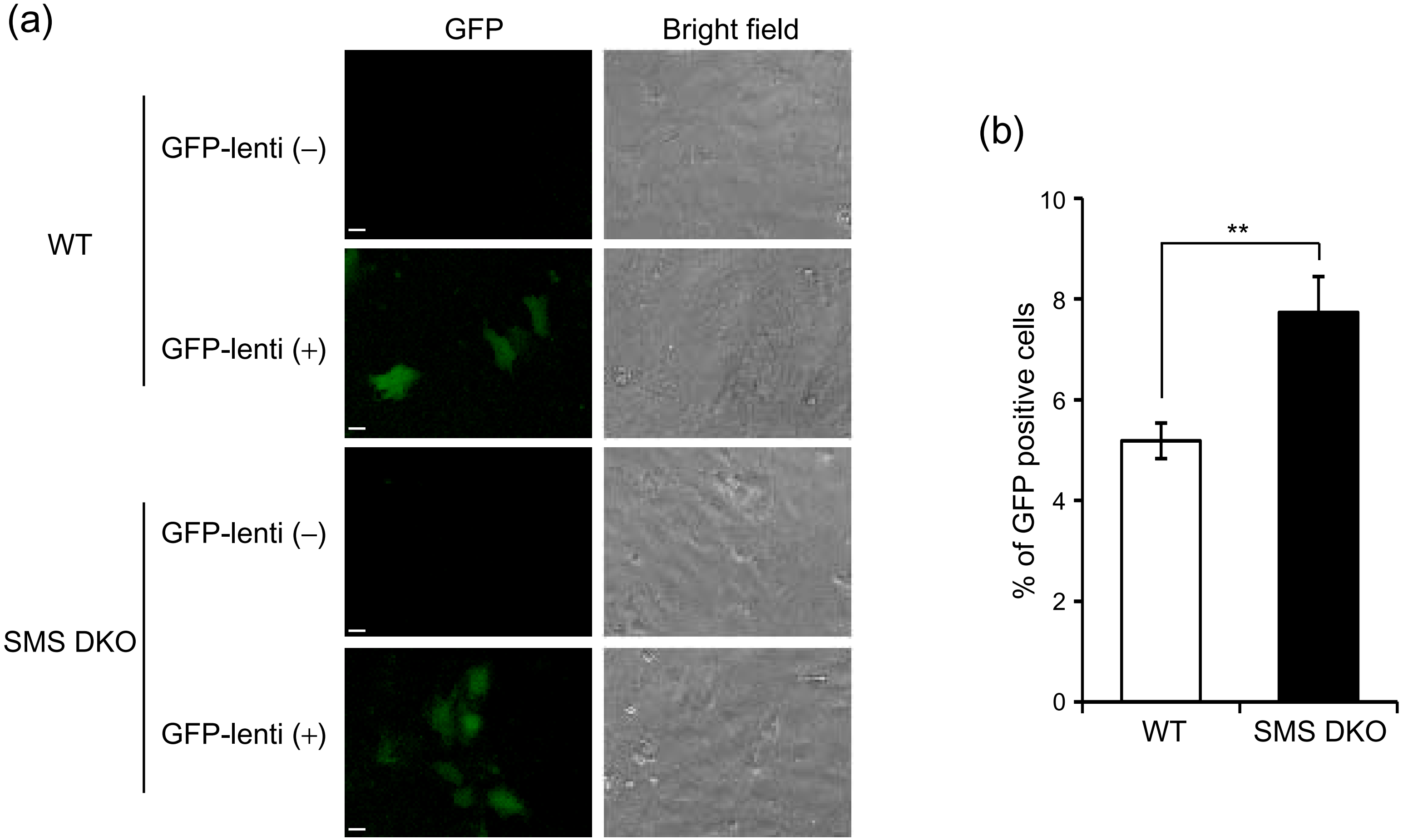
(a)
GFP
Bright field
WT
GFP-lenti (–)
GFP-lenti (+)
SMS DKO
GFP-lenti (–)
GFP-lenti (+)
(b)
% of GFP positive cells
10
8
6
4
2
0
**
WT
SMS DKO

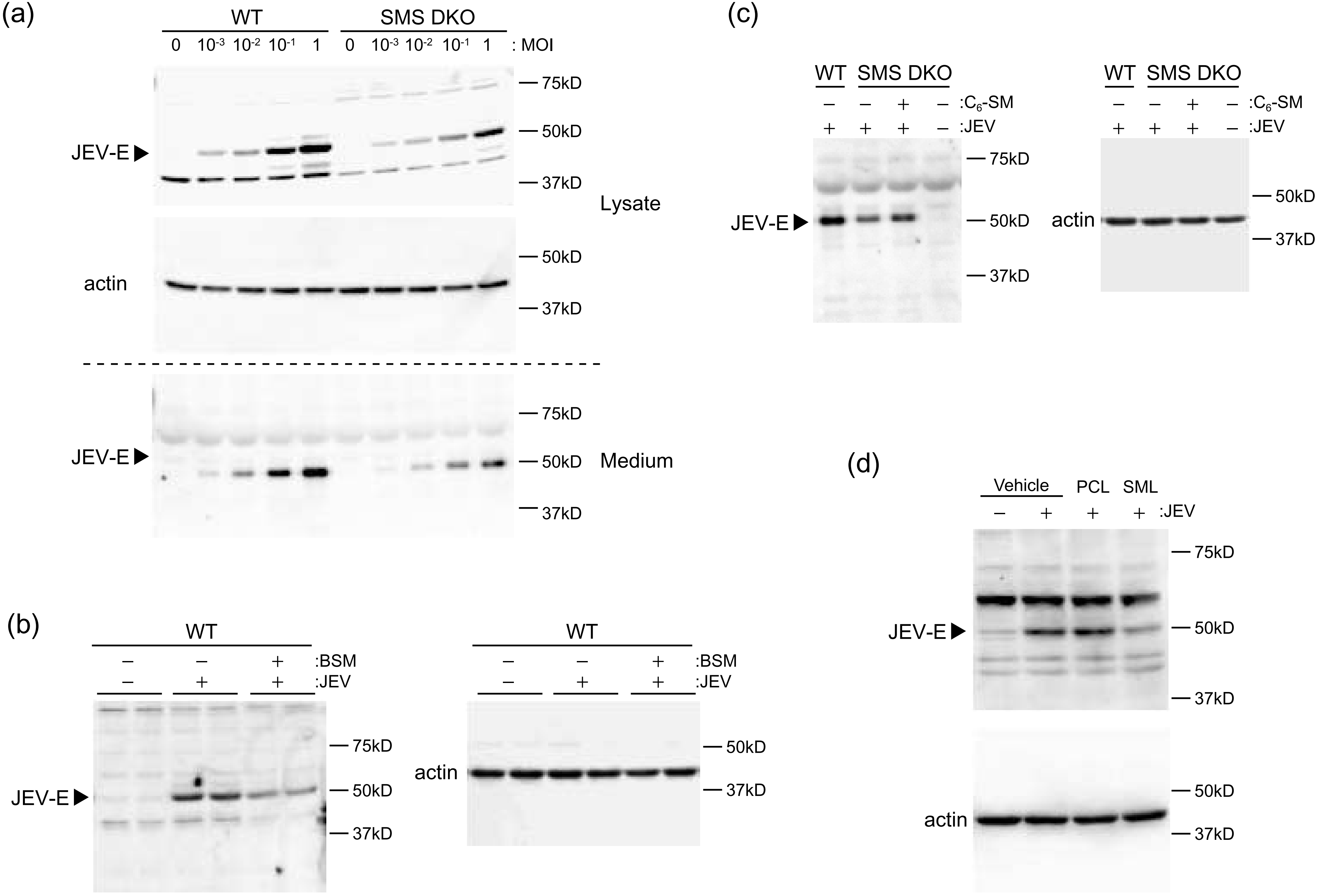
(a)
WT
SMS DKO
0 10-3 10-2 10-1 1 0 10-3 10-2 10-1 1 : MOI
JEV-E
75kD
50kD
37kD
Lysate
actin
50kD
37kD
JEV-E
75kD
50kD
37kD
Medium
(b)
WT
– – + :BSM
– + + :JEV
JEV-E
75kD
50kD
37kD
WT
– – + :BSM
– + + :JEV
actin
50kD
37kD
(c)
WT SMS DKO
– – + – :C6-SM
+ + + – :JEV
JEV-E
75kD
50kD
37kD
WT SMS DKO
– – + – :C6-SM
+ + + – :JEV
actin
50kD
37kD
(d)
Vehicle PCL SML
– + + + :JEV
JEV-E
75kD
50kD
37kD
actin
50kD
37kD

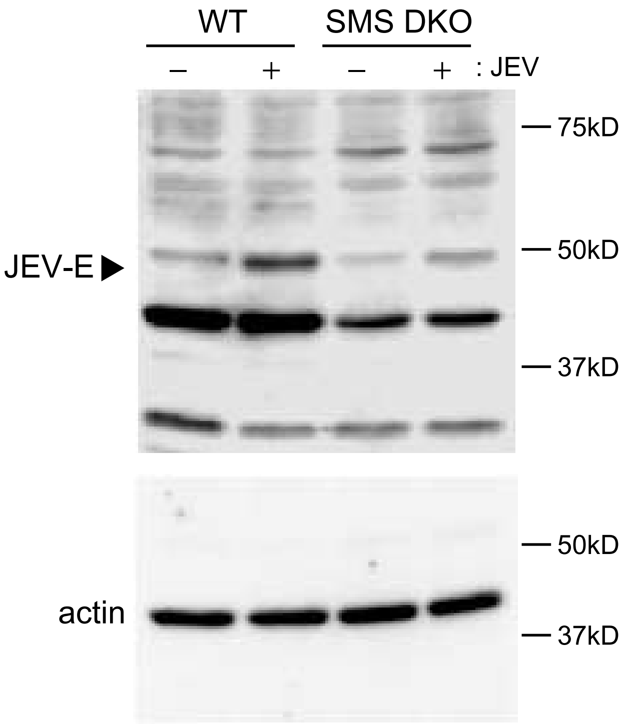
WT
SMS DKO
– + – + : JEV
75kD
50kD
37kD
JEV-E
actin
50kD
37kD

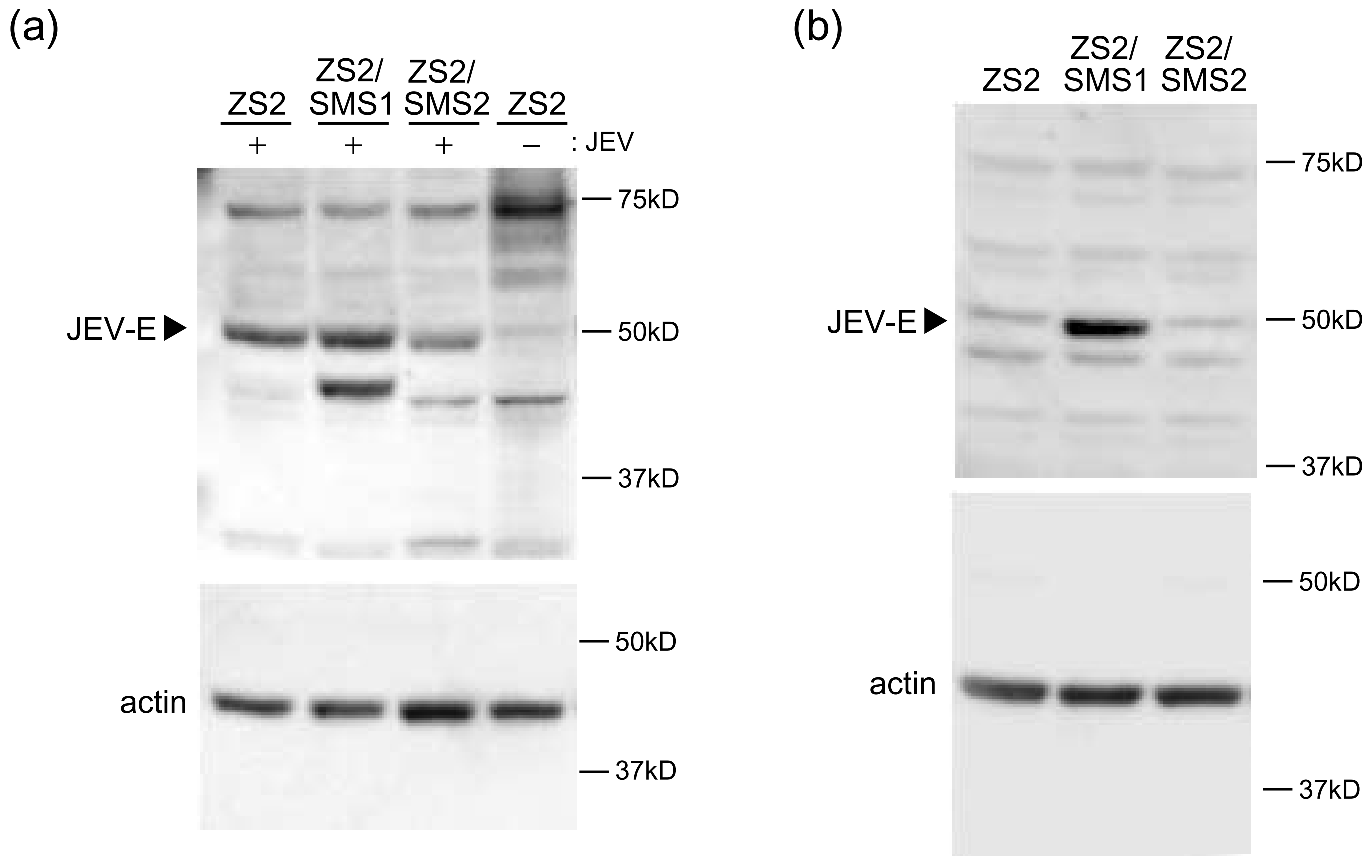

(a)
ZS2
ZS2/ SMS1
ZS2/ SMS2
ZS2
+
+
+
–
: JEV
75kD
50kD
37kD
JEV-E
50kD
37kD
actin
(b)
ZS2
ZS2/ SMS1
ZS2/ SMS2
75kD
50kD
37kD
JEV-E
50kD
37kD
actin

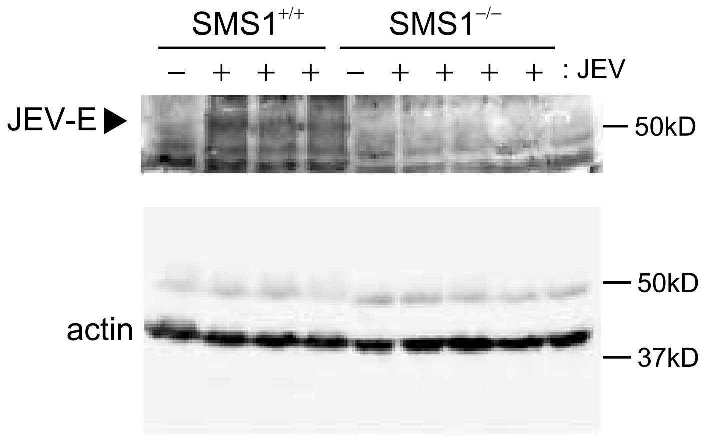
SMS1+/+
SMS1−/−
− + + + − + + + + : JEV
JEV-E
50kD
50kD
actin
37kD
